# Supplementary material for: NK cell spatial dynamics and IgA responses in gut-associated lymphoid tissues during SIV infections
Source: Commun Biol. 2022 Jul 7;5:674. doi: 10.1038/s42003-022-03619-y (PMC9262959; doi:10.1038/s42003-022-03619-y)
Supplement: Supplementary file 2 — Supplementary Information [file 42003_2022_3619_MOESM2_ESM.pdf]

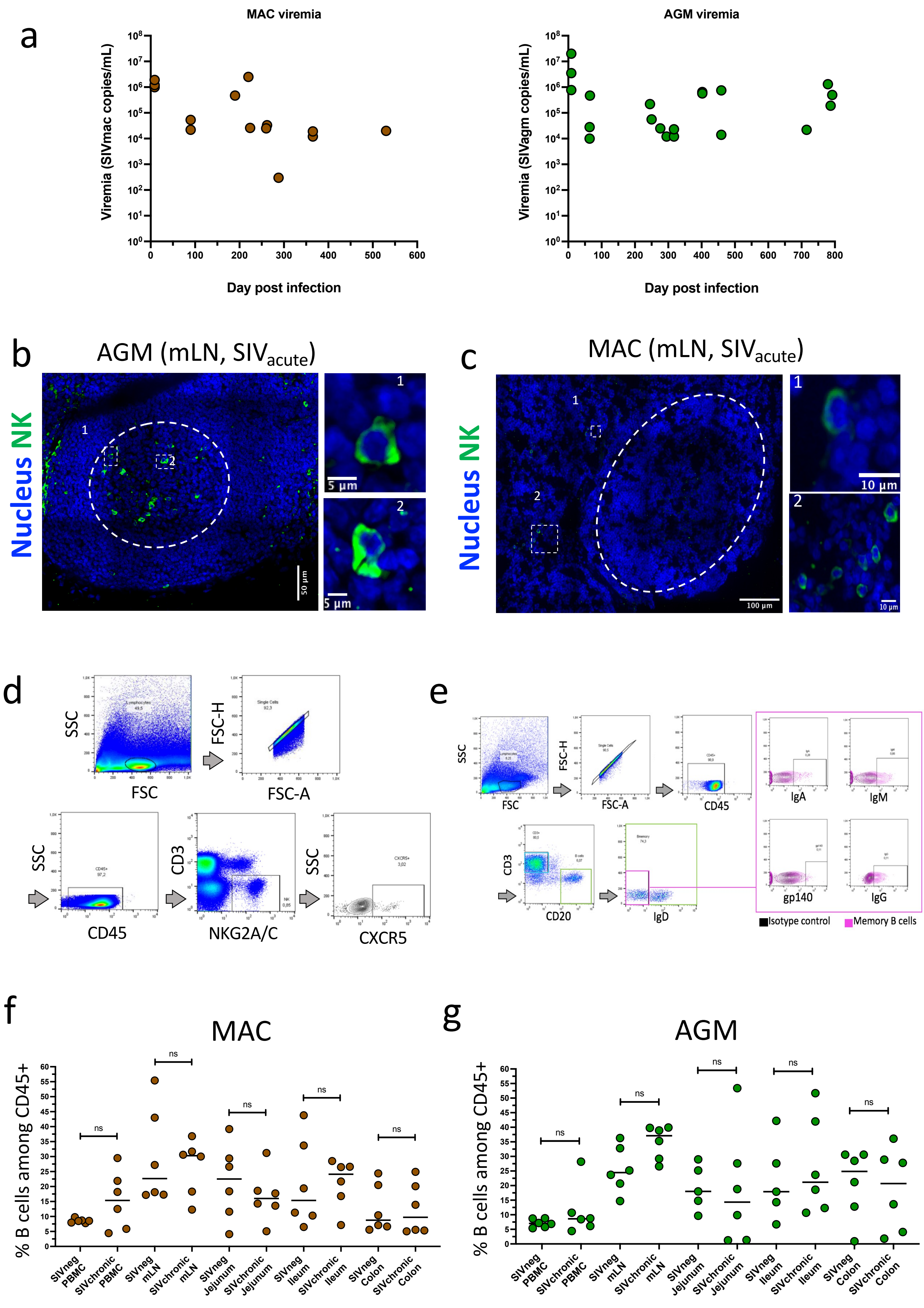

a
MAC viremia
AGM viremia
Viremia (SIVmac copies/mL)
Viremia (SIVagm copies/mL)
Day post infection
b
AGM (mLN, SIVacute)
Nucleus NK
5 µm
50 µm
c
MAC (mLN, SIVacute)
10 µm
100 µm
d
SSC
FSC
FSC-H
FSC-A
CD45
CD3
NKG2A/C
CXCR5
e
IgA
IgM
gp140
IgG
CD20
IgD
Isotype control
Memory B cells
f
MAC
g
AGM
% B cells among CD45+
ns
SIVneg PBMC
SIVchronic PBMC
SIVneg mLN
SIVchronic mLN
SIVneg Jejunum
SIVchronic Jejunum
SIVneg Ileum
SIVchronic Ileum
SIVneg Colon
SIVchronic Colon

Supplementary Figure 1: SIVagm and SIVmac CXCR5+ NK and B cell phenotype and viremia. a) Viremia levels for the 20 SIVagm-infected AGM and 14 SIVmac-infected MAC. The time points shown for each monkey represent the viral load at necropsy corresponding to the time of sample collection for this study. These data are described in detail in Supplementary Data 1-2. **b)** Immunofluorescence staining of NK cells in mesLN during acute SIVagm infection. Three animals per species were analyzed at day 9 p.i. (SIVacute). Nucleus is stained in blue, NK cells in green. The dash white circle delineates the B cell follicle area. **c)** Immunofluorescence staining of NK cells in mesLN during acute SIVmac infection. Three animals per species were analyzed at day 9 p.i. (SIVacute). Nucleus is stained in blue, NK cells in green. The dash white circle delineates the B cell follicle area. **d)** A dot plot showing CXCR5+ NK cells. NK cells in AGM were gated as usual for NHP as CD45+CD3-NKG2A/C+17. **e)** Gating strategy used for identifying IgG+/IgA+/IgM+/GP140+ memory B cells in MAC and AGM. **f)** Percentage of total B cells in distinct compartments (blood, mesLN, jejunum, ileum, and colon) in non-infected (SIVneg) and chronically SIVmac-infected MAC (SIVchronic). Six animals per species and per time points were analyzed. **g)** Percentage of total B cells in distinct compartments (blood, mesLN, jejunum, ileum, and colon) in non-infected (SIVneg) and chronically SIVagm-infected AGM (SIVchronic). Six animals per species and per time points were analyzed.

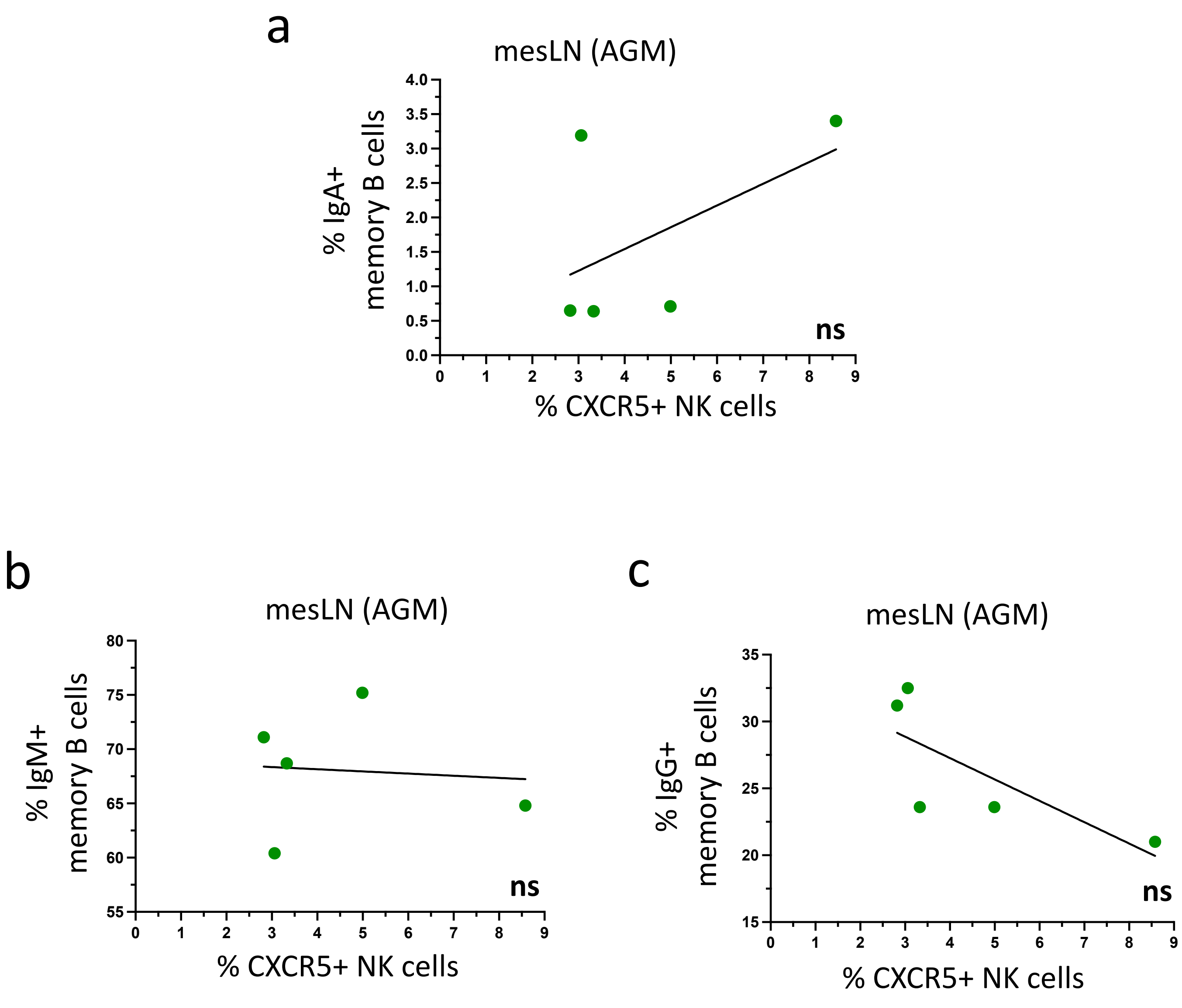
a
mesLN (AGM)
% IgA+ memory B cells
4.0
3.5
3.0
2.5
2.0
1.5
1.0
0.5
0.0
0
1
2
3
4
5
6
7
8
9
% CXCR5+ NK cells
ns
b
mesLN (AGM)
% IgM+ memory B cells
80
75
70
65
60
55
0
1
2
3
4
5
6
7
8
9
% CXCR5+ NK cells
ns
c
mesLN (AGM)
% IgG+ memory B cells
35
30
25
20
15
0
1
2
3
4
5
6
7
8
9
% CXCR5+ NK cells
ns

Supplementary Figure 2: IgA, IgM, and IgG correlation in the mesLN from chronically infected AGM. Correlation between CXCR5+ NK cells and **a)** IgA+, **b)** IgM+ and **c)** IgG+ memory B cell in mesLN from chronically SIVagm-infected AGM (dark green). Each green circle indicates an individual animal (n= 5 animals). Spearman r test was used (p-value≤0.05=*; p-value≤0.01=**; p-value≤0.001=***). Animals and time points of tissue collections are described in Supplementary Data 1-2.

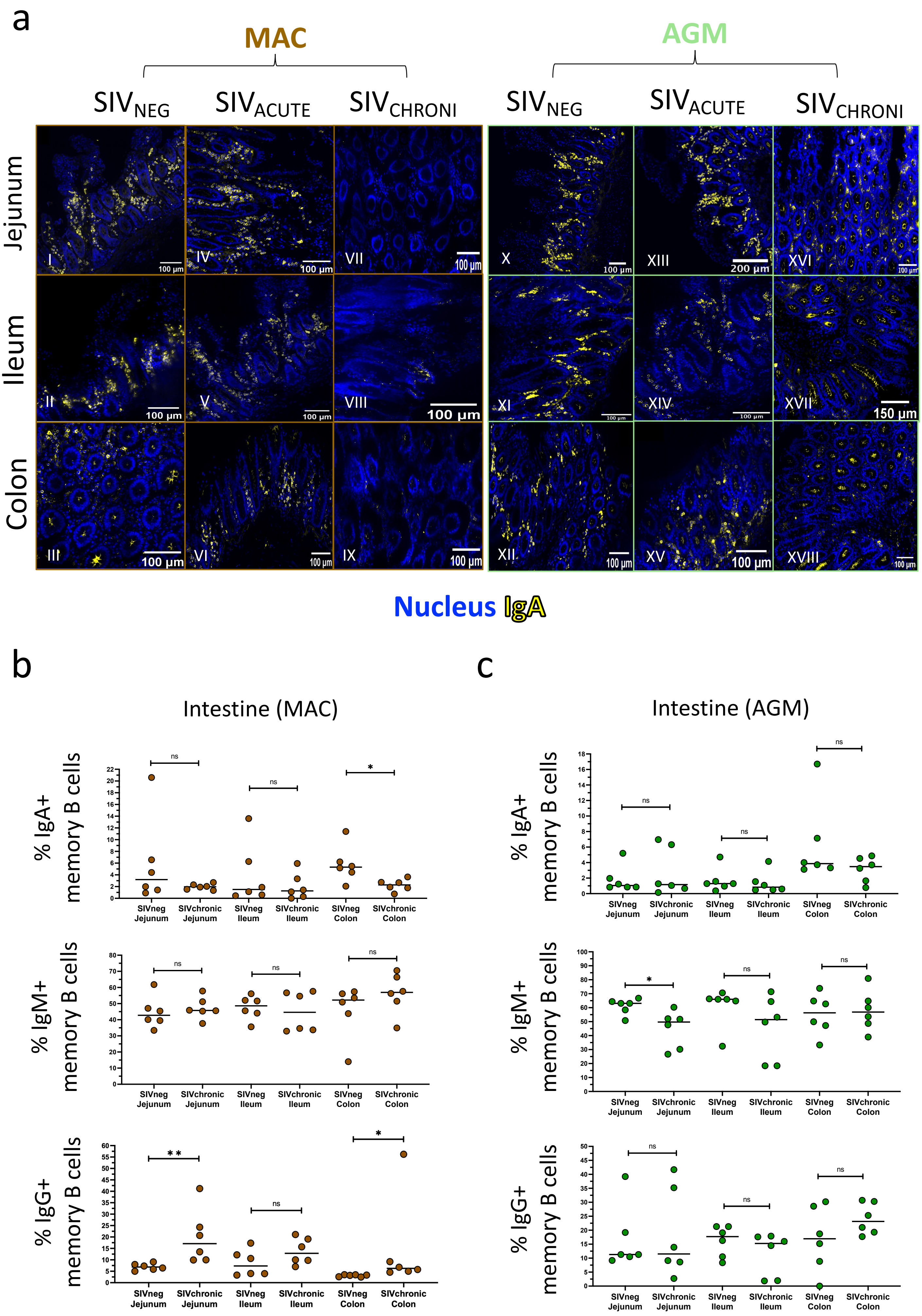
a
MAC
AGM
SIVNEG
SIVACUTE
SIVCHRONI
Jejunum
Ileum
Colon
Nucleus IgA
b
Intestine (MAC)
% IgA+ memory B cells
% IgM+ memory B cells
% IgG+ memory B cells
SIVneg Jejunum
SIVchronic Jejunum
SIVneg Ileum
SIVchronic Ileum
SIVneg Colon
SIVchronic Colon
c
Intestine (AGM)

Supplementary Figure 3: IgA, IgM and IgG production in the intestine during SIVagm and SIVmac infections. a) Immunofluorescence staining of IgA in the intestine (jejunum) from MAC (non-infected (SIVneg), at day 9 p.i. (SIVacute) and chronically SIVmac-infected (SIVchronic)) and AGM (non-infected (SIVneg), at day 9 p.i. (SIVacute) and chronically SIVagm-infected (SIVchronic)). Three non-infected MAC (ileum, colon), five non-infected MAC (jejunum) and three non-infected AGM (jejunum, ileum, colon) were analyzed. Three MAC (jejunum, ileum, colon) and three AGM (jejunum, ileum, colon) were analyzed at day 9 p.i. Three chronic MAC (colon), four chronic MAC (ileum), five chronic MAC (jejunum), four chronic AGM (ileum) and three chronic AGM (jejunum, colon) were analyzed. Representative sections for one animal per species are shown. **b-c)** Percentage of IgA+, IgM+ and IgG+ memory B cells in three compartments of the intestine (jejunum, ileum, and colon) from **b)** MAC (non-infected (SIVneg) and chronically SIVmac-infected (SIVchronic)) (n = 6 animals) and **c)** AGM (non-infected (SIVneg) and chronically SIVmac infected (SIVchronic)) (n = 6 animals). A nonparametric Mann-Whitney test (p-value≤0.05=*; pvalue≤0.01=**; p-value≤0.001=***) was used.

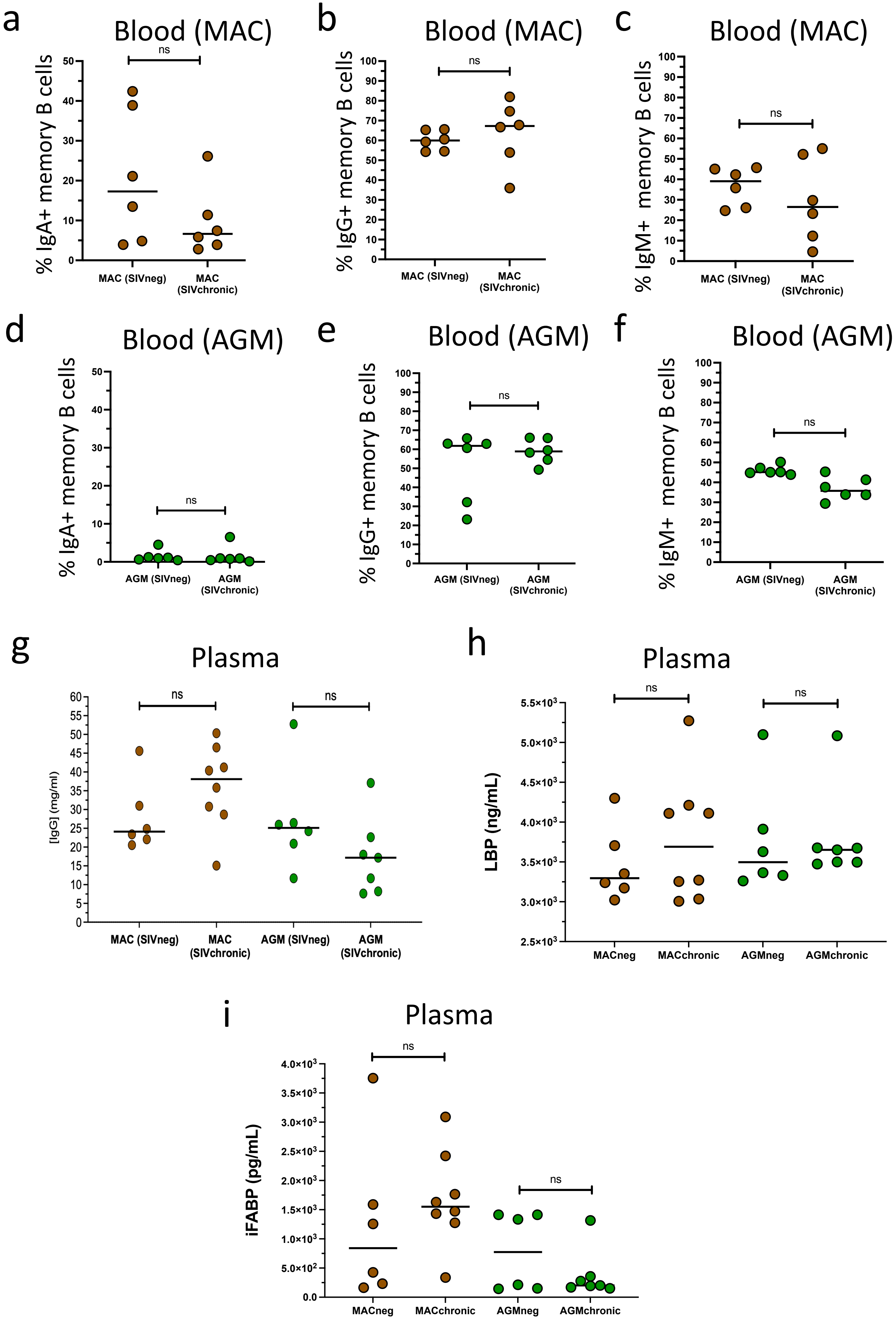
a
Blood (MAC)
% IgA+ memory B cells
MAC (SIVneg)
MAC (SIVchronic)
ns
b
Blood (MAC)
% IgG+ memory B cells
MAC (SIVneg)
MAC (SIVchronic)
ns
c
Blood (MAC)
% IgM+ memory B cells
MAC (SIVneg)
MAC (SIVchronic)
ns
d
Blood (AGM)
% IgA+ memory B cells
AGM (SIVneg)
AGM (SIVchronic)
ns
e
Blood (AGM)
% IgG+ memory B cells
AGM (SIVneg)
AGM (SIVchronic)
ns
f
Blood (AGM)
% IgM+ memory B cells
AGM (SIVneg)
AGM (SIVchronic)
ns
g
Plasma
[IgG] (mg/ml)
MAC (SIVneg)
MAC (SIVchronic)
AGM (SIVneg)
AGM (SIVchronic)
ns
h
Plasma
LBP (ng/mL)
MACneg
MACchronic
AGMneg
AGMchronic
ns
i
Plasma
iFABP (pg/mL)
MACneg
MACchronic
AGMneg
AGMchronic
ns

Supplementary Figure 4: Plasma titers of inflammatory and bacteria translocation markers. a-c) Percentages of IgA+, IgG+ and IgM+ memory B cells in blood from MAC (noninfected (SIVneg) and chronically SIVmac-infected (SIVchronic)) (n= 6 animals). A nonparametric Mann-Whitney test (p-value≤0.05=*; p-value≤0.01=**; p-value≤0.001=***) was used. **d-f)** Percentage of IgA+, IgG+ and IgM+ memory B cells in blood from AGM (non-infected (SIVneg) and chronically SIVagm-infected (SIVchronic)) (n= 6 animals). A nonparametric Mann-Whitney test (p-value≤0.05=*; p-value≤0.01=**; p-value≤0.001=***) was used. **g)** IgG titers in plasma from MAC and AGM (non-infected (SIVneg) and chronically SIV-infected (SIVchronic)) (n= 6-8 animals). A nonparametric Mann-Whitney test (p-value≤0.05=*; p-value≤0.01=**; p-value≤0.001=***) was used. **h)** Plasma titers of LBP and **i)** I-FABP from non-infected (SIVneg) and chronically SIV-infected (SIVchronic) MAC and AGM (n=6 non-infected animals per species, n= 8 chronic MAC and n= 7 chronic AGM). From Supplementary Figure a to Supplementary Figure c, a nonparametric Mann-Whitney test (p-value≤0.05=*; p-value≤0.01=**; p-value≤0.001=***) was used. Each dot represents an individual animal.

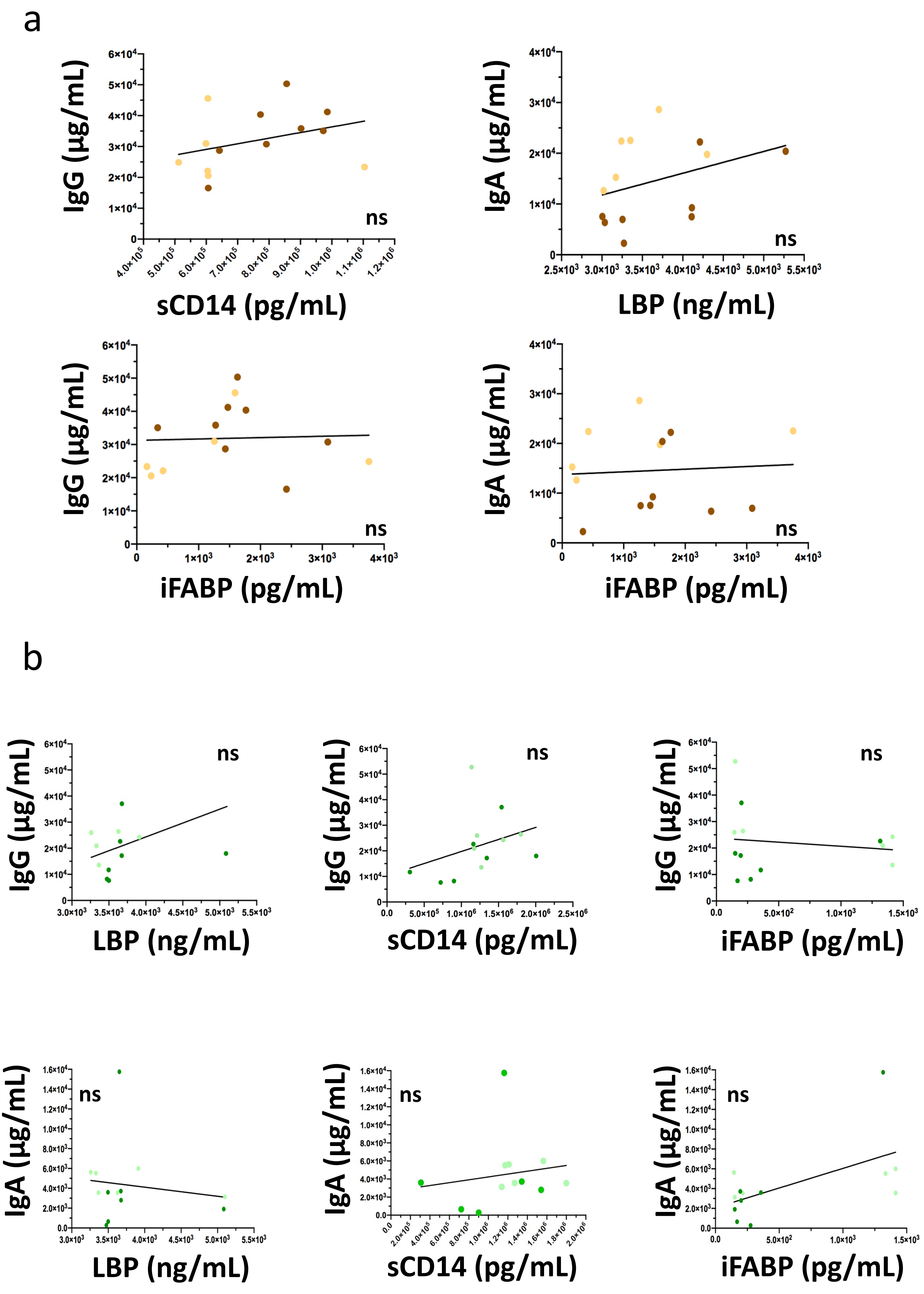
a
IgG (µg/mL)
sCD14 (pg/mL)
ns
IgA (µg/mL)
LBP (ng/mL)
ns
IgG (µg/mL)
iFABP (pg/mL)
ns
IgA (µg/mL)
iFABP (pg/mL)
ns
b
IgG (µg/mL)
LBP (ng/mL)
ns
IgG (µg/mL)
sCD14 (pg/mL)
ns
IgG (µg/mL)
iFABP (pg/mL)
ns
IgA (µg/mL)
LBP (ng/mL)
ns
IgA (µg/mL)
sCD14 (pg/mL)
ns
IgA (µg/mL)
iFABP (pg/mL)
ns

Supplementary Figure 5: Analyses of the correlations between plasmatic titers of IgG or IgA and soluble inflammation or microbial translocation markers in blood. a) Correlation between plasmatic IgG or IgA antibody levels and sCD14, LBP and iFABP in non-infected (light brown) and chronically SIV infected MAC (dark brown). The IgA versus sCD14 and IgG versus LBP panels are exhibited in Figure 3c-d. **b)** Correlation between plasmatic IgG and IgA antibody levels and sCD14, LBP and iFABP in non-infected (light green) and chronically SIV infected (dark green) AGM. Each dot indicates an individual animal (n=6 non-infected animals per species, n= 8 chronic MAC and n= 7 chronic AGM). Spearman r test was used.

a

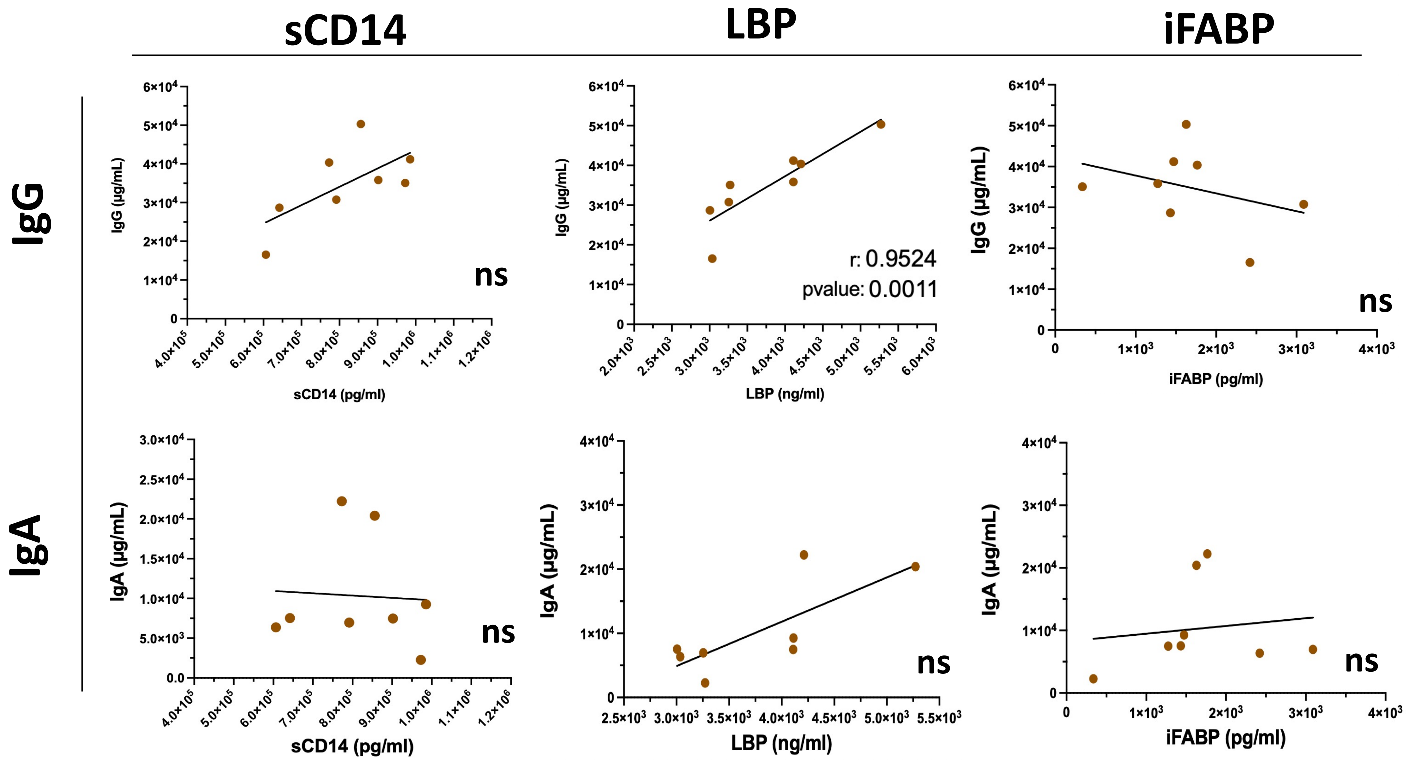
sCD14
LBP
iFABP
IgG
IgA
IgG (µg/mL)
IgA (µg/mL)
sCD14 (pg/ml)
LBP (ng/ml)
iFABP (pg/ml)
r: 0.9524
pvalue: 0.0011
ns

b

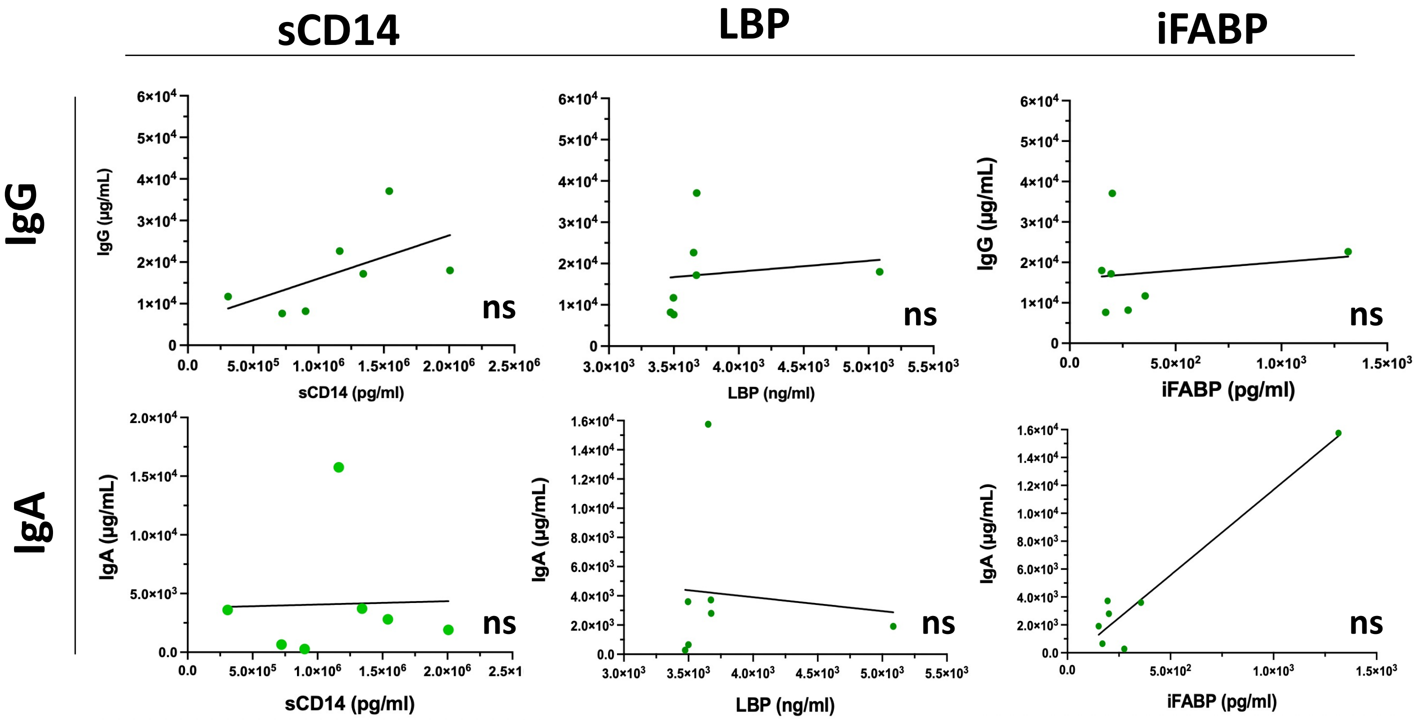
sCD14
LBP
iFABP
IgG
IgA
IgG (µg/mL)
IgA (µg/mL)
sCD14 (pg/ml)
LBP (ng/ml)
iFABP (pg/ml)
ns

Supplementary Figure 6: Analyses of the correlations between plasmatic titers of IgG or IgA and soluble inflammation or microbial translocation markers in blood from infected animals. a) Correlation between plasmatic IgG or IgA antibody levels and sCD14, LBP and iFABP in chronically SIV-mac infected MAC. **b)** Correlation between plasmatic IgG or IgA antibody levels and sCD14, LBP and iFABP in chronically SIV infected AGM. Each dot indicates an individual animal (n= 8 chronic MAC and n= 7 chronic AGM). Spearman r test was used.

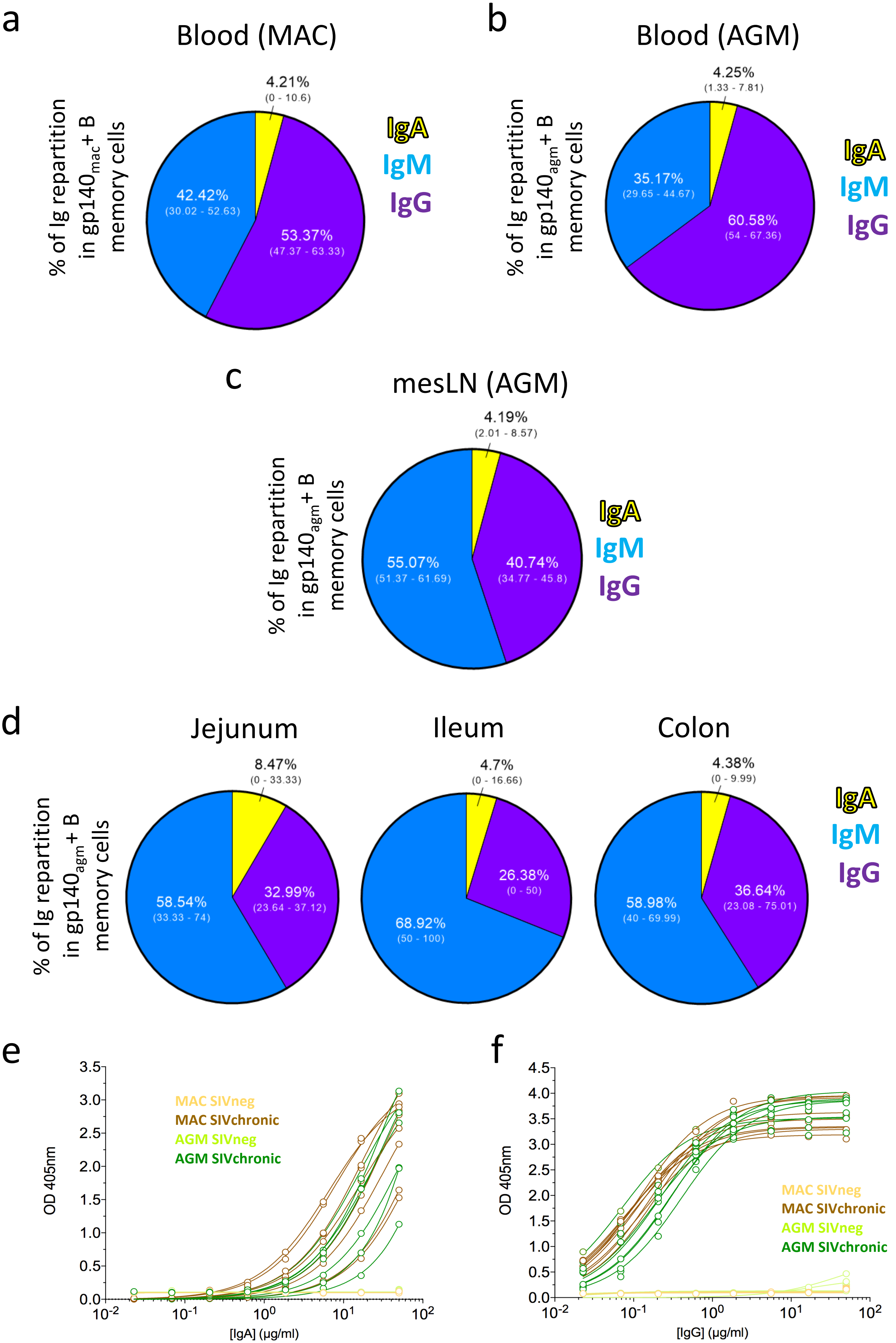
a
Blood (MAC)
% of Ig repartition in gp140mac+ B memory cells
4.21%
(0 - 10.6)
42.42%
(30.02 - 52.63)
53.37%
(47.37 - 63.33)
IgA
IgM
IgG
b
Blood (AGM)
% of Ig repartition in gp140agm+ B memory cells
4.25%
(1.33 - 7.81)
35.17%
(29.65 - 44.67)
60.58%
(54 - 67.36)
IgA
IgM
IgG
c
mesLN (AGM)
% of Ig repartition in gp140agm+ B memory cells
4.19%
(2.01 - 8.57)
55.07%
(51.37 - 61.69)
40.74%
(34.77 - 45.8)
IgA
IgM
IgG
d
% of Ig repartition in gp140agm+ B memory cells
Jejunum
8.47%
(0 - 33.33)
58.54%
(33.33 - 74)
32.99%
(23.64 - 37.12)
Ileum
4.7%
(0 - 16.66)
68.92%
(50 - 100)
26.38%
(0 - 50)
Colon
4.38%
(0 - 9.99)
58.98%
(40 - 69.99)
36.64%
(23.08 - 75.01)
IgA
IgM
IgG
e
OD 405nm
[IgA] (µg/ml)
MAC SIVneg
MAC SIVchronic
AGM SIVneg
AGM SIVchronic
f
OD 405nm
[IgG] (µg/ml)
MAC SIVneg
MAC SIVchronic
AGM SIVneg
AGM SIVchronic

Supplementary Figure 7: IgA, IgM and IgG repartition in GP140+ memory B cells. A-E) Pie representation of median percentages of GP140+ IgA+, IgG+ and IgM+ memory B cells. The range (min-max) is indicated in brackets. **a)** GP140+ IgA+, IgG+ and IgM+ memory B cells from blood in chronically SIVmac-infected MAC (n=6 animals). **b)** GP140+ IgA+, IgG+ and IgM+ memory B cells from blood in chronically SIVagm-infected AGM (n=6 animals). **c)** GP140+ IgA+, IgG+ and IgM+ memory B cells from mesLN in chronically SIVagm-infected AGM (n=6 animals). **d)** GP140+ IgA+, IgG+ and IgM+ memory B cells from intestine (jejunum, ileum, and colon) of chronically SIVagm-infected AGM (n=6 animals). **e)** Binding of plasmatic SIV-specific IgA antibodies to trimeric GP140 proteins measured by ELISA. The green dotted line corresponds to the AGM, light green represents non-infected monkeys and dark green represents chronically infected monkeys. The brown dotted line corresponds to the MAC, light brown represents non-infected monkeys and dark brown represents chronically infected monkeys (n=6 non-infected animals per species, n= 8 chronic MAC and n= 7 chronic AGM). **f)** ELISA graphs show the binding of plasmatic SIV-specific IgG antibodies to trimeric GP140 proteins. The green dotted line corresponds to the AGM, light green represents non-infected monkeys and dark green represents chronically infected monkeys. The brown dotted line corresponds to the MAC, light brown represents non-infected monkeys and dark brown represents chronically infected monkeys (n=6 non-infected animals per species, n= 8 chronic MAC and n= 7 chronic AGM).

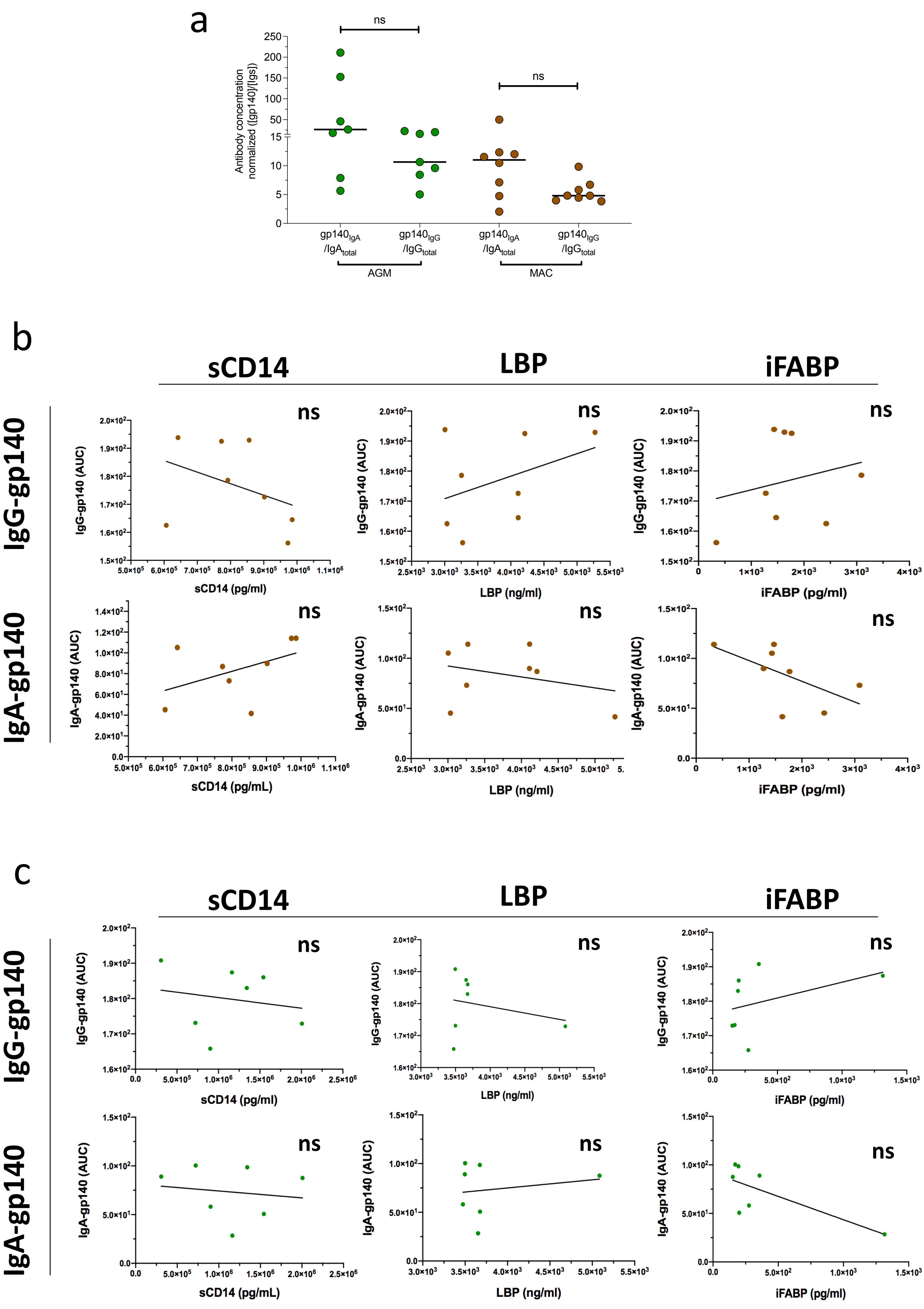
a
ns
ns
Antibody concentration normalized ([gp140]/[Igs])
250
200
150
100
50
15
10
5
0
gp140IgA /IgAtotal
gp140IgG /IgGtotal
gp140IgA /IgAtotal
gp140IgG /IgGtotal
AGM
MAC
b
sCD14
LBP
iFABP
IgG-gp140
IgA-gp140
ns
IgG-gp140 (AUC)
IgA-gp140 (AUC)
sCD14 (pg/ml)
sCD14 (pg/mL)
LBP (ng/ml)
iFABP (pg/ml)
c
sCD14
LBP
iFABP
IgG-gp140
IgA-gp140
ns
IgG-gp140 (AUC)
IgA-gp140 (AUC)
sCD14 (pg/ml)
sCD14 (pg/mL)
LBP (ng/ml)
iFABP (pg/ml)

Supplementary Figure 8: Search for potential correlations between SIVagm and SIVmac Env-specific IgA and IgG in blood and markers of microbial translocation and systemic inflammation. a) Ratio of GP-140 specific antibody concentration on total antibody concentration in plasma from AGM (7 non-infected and 7 chronically infected animals) (green dots indicate each an individual AGM) and MAC (8 non-infected and 8 chronically infected animals; each brown dot indicates an individual animal). A nonparametric Mann-Whitney test (p-value≤0.05=*; p-value≤0.01=**; p-value≤0.001=***) was used. **b)** Correlation between plasma titers of GP140-specific IgG or IgA and sCD14, LBP or I-FABP concentrations in plasma of SIVmac infected MAC (brown). Each dot indicates an individual animal (n= 8 chronic MAC). **c)** Correlation between plasma titers of GP140-specific IgG or IgA and sCD14, LBP or I-FABP concentrations in plasma of SIVagm infected AGM (green). Each dot indicates an individual animal (n= 7 chronic AGM). Spearman r test was used.
