# Supplementary material for: NK cell spatial dynamics and IgA responses in gut-associated lymphoid tissues during SIV infections
Source: Commun Biol. 2022 Jul 7;5:674. doi: 10.1038/s42003-022-03619-y (PMC9262959; doi:10.1038/s42003-022-03619-y)
Supplement: Supplementary file 3 — Description of Additional Supplementary Files [file 42003_2022_3619_MOESM3_ESM.pdf]

Description of Additional Supplementary Files

File name: Supplementary Data 1

Description: Tissue collections and SIV infection profiles in the animals. Table showing sample collection and virological parameters of African Green 896 Monkeys non-infected or infected with SIVagm.sab92018.

File name: Supplementary Data 2

Description: Tissue collections and SIV infection profiles in the animals. Table showing sample collection and virological parameters of 26 cynomolgus macaques non-infected or infected 898 with SIVmac251.

File name: Supplementary Data 3

Description: Antibodies used for flow cytometry staining. Table showing antibodies used to gating immunoglobulin, SIV specificity (GP140) on memory B cells and CXCR5 on NK cells in different tissues from both species (AGM, MAC).

File name: Supplementary Data 4

Description: Antibodies used for microscope staining. Table showing antibodies used to identify NK cells and IgA+ cells in different tissues from both species (AGM, MAC).

File name: Supplementary Data 5

Description: CD89 gene expression profiles in NK cells from lymph node and blood during chronic SIVagm-infection in AGM. Genome-wide transcriptomes were used to evaluate CD89 gene expression (FCAR) in distinct NK cell subpopulations: CXCR5+ NK from LN (LNC), CD16-NKG2Alow NK from LN (LN-0), CD16-NKG2A+ NK from LN (LN- 1), CD16+NKG2Alow/+ NK from LN (LN-23), CD16-NKG2Alow NK from blood (PB-0), CD16-NKG2A+ NK from blood (PB-1), CD16+NKG2A+ NK from LN (PB-2) and CD16+NKG2Alow NK from LN (PB-3). The isolation of these NK cell populations has been described elsewhere. CD89 gene expressions were compared to that of NKG2A gene expression (KLRC1) and IL-10 gene expression (IL10) in these same cells. The blood and LN from three SIVagm chronically infected AGM were analyzed (n=3 animals, notified as 83, 93 and 101), as previously reported. Source data been deposited in the Gene Expression Omnibus database by Huot et al. Nat. Med. 2021; the accession number is GSE140600.

File name: Supplementary Data 6

Description: Source data underlying the graphs and charts presented in the main figures.
