# Supplementary material for: NK cell spatial dynamics and IgA responses in gut-associated lymphoid tissues during SIV infections
Source: Commun Biol. 2022 Jul 7;5:674. doi: 10.1038/s42003-022-03619-y (PMC9262959; doi:10.1038/s42003-022-03619-y)
Supplement: Supplementary file 10 — Reporting Summary [file 42003_2022_3619_MOESM10_ESM.pdf]

nature research

Corresponding author(s): DBPR, COMMSBIO-21-1581

Last updated by author(s): Feb 22, 2022

Reporting Summary

Nature Research wishes to improve the reproducibility of the work that we publish. This form provides structure for consistency and transparency in reporting. For further information on Nature Research policies, see our Editorial Policies and the Editorial Policy Checklist.

Statistics

For all statistical analyses, confirm that the following items are present in the figure legend, table legend, main text, or Methods section.

n/a	Confirmed	
☐	☒	The exact sample size (n) for each experimental group/condition, given as a discrete number and unit of measurement
☐	☒	A statement on whether measurements were taken from distinct samples or whether the same sample was measured repeatedly
☐	☒	The statistical test(s) used AND whether they are one- or two-sided<br>*Only common tests should be described solely by name; describe more complex techniques in the Methods section.*
☒	☐	A description of all covariates tested
☐	☒	A description of any assumptions or corrections, such as tests of normality and adjustment for multiple comparisons
☐	☒	A full description of the statistical parameters including central tendency (e.g. means) or other basic estimates (e.g. regression coefficient) AND variation (e.g. standard deviation) or associated estimates of uncertainty (e.g. confidence intervals)
☐	☒	For null hypothesis testing, the test statistic (e.g. F, t, r) with confidence intervals, effect sizes, degrees of freedom and P value noted<br>*Give P values as exact values whenever suitable.*
☒	☐	For Bayesian analysis, information on the choice of priors and Markov chain Monte Carlo settings
☒	☐	For hierarchical and complex designs, identification of the appropriate level for tests and full reporting of outcomes
☐	☒	Estimates of effect sizes (e.g. Cohen's d, Pearson's r), indicating how they were calculated

Our web collection on statistics for biologists contains articles on many of the points above.

Software and code

Policy information about availability of computer code

Data collection	Flow Cytometry data was collected using FACS Diva sotfware on a LSR II (BD Bioscience). RNA-seq data was collected on an Illumina HiSeq 3000 system. Plasma viremia was collected using an Applied Biosystems 7500 Real-Time PCR. Images were collected using CellVoyager CV1000 (Yokagawa).
Data analysis	Flow cytometry acquisitions were done on a LSR II (BD Biosciences) using Diva software version (6.1.3). The data were further analyzed using FlowJo 10.8.1 software (FlowJo, LLC, Ashland, OR, USA). Statistical analyses were performed using GraphPad Prism 9. Images were analyzed by ImageJ (Fiji). Biotinylated SIV GP140 trimers were measured using a NanoDrop 2000 instrument (Thermo Fisher Scientific). All ELISA experiments were done in duplicates at room temperature using HydroSpeed microplate washer and Sunrise microplate absorbance reader (Tecan Mannedorf).<br>Bioinformatic analyses were performed using the RNA-seq pipeline from Sequana69. Reads were cleaned of adapter sequences, and low-quality sequences were removed using cutadapt version 1.11. Only sequences R25 nucleotides (nt) in length were considered for further analysis. STAR version 2.5.0a, with default parameters, was used for alignment on the reference genome (Chlorocebus sabaeus, from Ensembl release 90). Genes were counted using feature counts version 1.4.6-p3 from Subreads package (parameters: -t gene, -g ID and -s 1). Data were analyzed using R version 3.4.3 and the Bioconductor package DESeq2 version 1.18.1.

For manuscripts utilizing custom algorithms or software that are central to the research but not yet described in published literature, software must be made available to editors and reviewers. We strongly encourage code deposition in a community repository (e.g. GitHub). See the Nature Research guidelines for submitting code & software for further information.

Data

Policy information about availability of data

All manuscripts must include a data availability statement. This statement should provide the following information, where applicable:

- Accession codes, unique identifiers, or web links for publicly available datasets
- A list of figures that have associated raw data
- A description of any restrictions on data availability

Deep sequencing data have been obtained from the Gene Expression Omnibus database; the accession number is GSE140600. https://www.ncbi.nlm.nih.gov/geo/query/acc.cgi?acc=GSE140600. The Table in the manuscript has been associated to the raw data file. The authors declare that all data of this study are available from the authors upon request.

Field-specific reporting

Please select the one below that is the best fit for your research. If you are not sure, read the appropriate sections before making your selection.

☒ Life sciences ☐ Behavioural & social sciences ☐ Ecological, evolutionary & environmental sciences

For a reference copy of the document with all sections, see nature.com/documents/nr-reporting-summary-flat.pdf

Life sciences study design

All studies must disclose on these points even when the disclosure is negative.

Sample size	The sample size varied between 3 and 8 monkeys per group (n = 6 in most experiments), chosen according to the tripartite harmonized International Council for Harmonization of Technical Requirements for Pharmaceuticals for Human Use (ICH) Guideline on Methodology (previously coded Q2B).
Data exclusions	No data were excluded from the analyses.
Replication	Plasma viremia was performed with three replicates (three independent experiments for each condition). We did not observe any major variation with all the replicates. For the other assays, we only used biological replica (ie distinct animals). No other attempts were made at replication due to the limitation in volume of samples, and also duration of the in vivo study (2 years), substantial costs associated with animal acquisition, per diem charges, and surgical costs.
Randomization	Sample collection and analyses were performed in random order.
Blinding	The investigators were not blinded while the animal handlers were blinded to group allocation.

Reporting for specific materials, systems and methods

We require information from authors about some types of materials, experimental systems and methods used in many studies. Here, indicate whether each material, system or method listed is relevant to your study. If you are not sure if a list item applies to your research, read the appropriate section before selecting a response.

Materials & experimental systems

n/a	Involved in the study	
☐	☒	Antibodies
☒	☐	Eukaryotic cell lines
☒	☐	Palaeontology and archaeology
☐	☒	Animals and other organisms
☒	☐	Human research participants
☒	☐	Clinical data
☒	☐	Dual use research of concern

Methods

n/a	Involved in the study	
☒	☐	ChIP-seq
☐	☒	Flow cytometry
☒	☐	MRI-based neuroimaging

Antibodies

Antibodies used	Described in supplementary table 2 and supplementary table 3.
Validation	Described in supplementary table 2 and supplementary table 3.

Animals and other organisms

Policy information about studies involving animals; ARRIVE guidelines recommended for reporting animal research

Laboratory animals	At the inclusion in the study the average weight of the monkeys was between 3 and 6 kg. All monkeys were young adults with an average age of 3–5 years at inclusion. Both males and females were used (60% females and 40% males for each species). Because H6 haplotypes are notably associated with viral control in cynomolgus macaques, macaques with H6 haplotype were excluded from this study.
Wild animals	Study did not involve wild animals.
Field-collected samples	Study did not involve field-collected samples.
Ethics oversight	The AGM and MAC were housed in IDMIT infrastructure facilities (CEA, Fontenay-aux-Roses, France) under animal facility authorization #D92-032-02 (Prefecture des Hauts de Seine, France) and in compliance with European Directive 2010/63/EU, the French regulations and the Standards for Human Care and Use of Laboratory Animals, of the Office for Laboratory Animal Welfare (OLAW, assurance number #A5826-01, US). The study was approved by the the institutional ethical committee "Comité d'Ethique en Expérimentation Animale du Commissariat à l'Energie Atomique et aux Energies Alternatives" (CEtEA #44). Monitoring of the monkeys was under the supervision of the veterinarians in charge of the animal facilities. Animal experimental protocols were approved by the Ethical Committee of Animal Experimentation (CETEA-DSV, IDF, France; Notification 12-098). The samples from the animals included here were from animals purchased and used for other studies. These studies were approved and accredited under statement numbers A16-016, A17-059, A12-006, A17-044, A13-005 and A15-035 by the ethics committee, registered and authorized under Number 44 at the French Ministry of Education and Research with the reference numbers APAFIS#2453-2015102713323361, APAFIS#4442-2016030818243239 and APAFIS# 11236-2017091214402801 and APAFIS#319-2015031314518254.02. Animals were handled by veterinarians in accordance with national regulations (CEA Permit Number A 92-32-02) and the European Directive (2010/63, recommendation no. 9) and in compliance with the Standards for Human Care and Use of Laboratory of the Office for Laboratory Animal Welfare (OLAW, USA) under OLAW Assurance number #A5826-01. Animals were housed in ASL3 confinement in adjoining individual cages allowing social interactions, and maintained under controlled conditions with respect to humidity, temperature, and light (12 hours light/12 hours dark cycles). Water was available ad libitum. Animals were monitored and fed once or twice daily commercial monkey chow and fruit by trained personnel. Environmental enrichment was provided including toys, novel foodstuffs, and music under the supervision of the CEA Animal Welfare Body. Experimental procedures (animal handling, viral inoculations, and samplings) were conducted after sedation with ketamine chlorhydrate (Rhone-Merieux, Lyon, France, 10 mg/kg). Tissues were collected at necropsy: animals were sedated with ketamine chlorhydrate 10 mg/kg) then humanely euthanized by intravenous injection of 180 mg/kg sodium pentobarbital.

Note that full information on the approval of the study protocol must also be provided in the manuscript.

Flow Cytometry

Plots

Confirm that:

☒ The axis labels state the marker and fluorochrome used (e.g. CD4-FITC).

☒ The axis scales are clearly visible. Include numbers along axes only for bottom left plot of group (a 'group' is an analysis of identical markers).

☒ All plots are contour plots with outliers or pseudocolor plots.

☒ A numerical value for number of cells or percentage (with statistics) is provided.

Methodology

Sample preparation	Whole venous blood was collected in ethylenediaminetetraacetic acid (EDTA) tubes. Peripheral blood mononuclear cells (PBMCs) were isolated by Ficoll density-gradient centrifugation. Biopsies of peripheral lymph nodes (pLN) were performed by excision. Other tissues were collected at autopsy. After careful removal of adhering connective and fat tissues, LN and spleen cells were dissociated using the gentlemacS™ Dissociator technology (Miltenyi Biotec, Germany). The cell suspension was subsequently filtered through 100- and 40-um cell strainers, and cells were washed with cold phosphate-buffered saline (PBS). Cells were either immediately stained for flow cytometry or cryopreserved in 90% foetal bovine serum (FBS) and 10% dimethyl sulfphoxide (DMSO) and stored in liquid nitrogen before use.
Instrument	Flow cytometry acquisitions were done on a LSR II (BD Biosciences).
Software	The data were analysed using Diva (software version 6.1.3, build 2009 05 13 13 29). The data were further analyzed using FlowJo (10.8.1 software, LLC, Ashland, OR, USA).
Cell population abundance	Described in supplemental figure 1.
Gating strategy	Described in supplemental figure 1.

☒ Tick this box to confirm that a figure exemplifying the gating strategy is provided in the Supplementary Information.
